# Differential Activity of *Drosophila* Hox Genes Induces Myosin Expression and Can Maintain Compartment Boundaries

**DOI:** 10.1371/journal.pone.0057159

**Published:** 2013-02-25

**Authors:** Jesús R. Curt, Luis F. de Navas, Ernesto Sánchez-Herrero

**Affiliations:** Centro de Biología Molecular Severo Ochoa, Consejo Superior de Investigaciones Científicas and Universidad Autónoma de Madrid, Madrid, Spain; Instituto Gulbenkian de Ciência, Portugal

## Abstract

Compartments are units of cell lineage that subdivide territories with different developmental potential. In *Drosophila*, the wing and haltere discs are subdivided into anterior and posterior (A/P) compartments, which require the activity of Hedgehog, and into dorsal and ventral (D/V) compartments, needing Notch signaling. There is enrichment in actomyosin proteins at the compartment boundaries, suggesting a role for these proteins in their maintenance. Compartments also develop in the mouse hindbrain rhombomeres, which are characterized by the expression of different Hox genes, a group of genes specifying different structures along their main axis of bilaterians. We show here that the *Drosophila* Hox gene *Ultrabithorax* can maintain the A/P and D/V compartment boundaries when Hedgehog or Notch signaling is compromised, and that the interaction of cells with and without *Ultrabithorax* expression induces high levels of non-muscle myosin II. In the absence of *Ultrabithorax* there is occasional mixing of cells from different segments. We also show a similar role in cell segregation for the *Abdominal-B* Hox gene. Our results suggest that the juxtaposition of cells with different Hox gene expression leads to their sorting out, probably through the accumulation of non-muscle myosin II at the boundary of the different cell territories. The increase in myosin expression seems to be a general mechanism used by Hox genes or signaling pathways to maintain the segregation of different groups of cells.

## Introduction

During animal development there is a progressive subdivision of the organism into distinct groups of cells that will form different organs and structures. In this process, the cells normally acquire different cellular affinities, which allow both to keep a coherent group of cells with the same fate and to distinguish them from surrounding cells with different identity [1].

The development of the *Drosophila* wing imaginal disc is a good model to study cell segregation. Wing and haltere imaginal discs are subdivided, early in development, into an anterior (A) and a posterior (P) compartment [2]. The selector gene *engrailed* (*en*) is expressed in the P compartment and induces the expression of the Hedgehog (Hh) signaling molecule. Cells from the P compartment, transcribing *en* and *hh*, do not mix with cells from the A compartment, lacking the expression of both genes. The boundary separating the two compartments forms a straight border, the line of minimal contact, named the antero-posterior (A/P) compartment boundary [2]–[4].

This strict lineage segregation can be compromised in two ways. First, posterior cells lacking *en* (and its cognate gene *invected*), can penetrate into the A compartment [3], [5]; reciprocally, if *en* is ectopically expressed in anterior cells, they can move to the P compartment [3]. Second, anterior cells mutant for *smoothened* (*smo*), an obligatory component of the Hh signaling pathway, can cross into the P compartment [6], [7]. Although a complete mixing of A and P cells requires changes in the activity of both En and the Hh pathway, eliminating the response to the Hh signal causes a more complete response and predominates over the mechanism depending on changes in *en* [3].

Wing and haltere imaginal disc are further subdivided into dorsal (D) and ventral (V) compartments. Dorsal cells transcribe *apterous* (*ap*), which regulates the expression of *Serrate* (*Ser*), a ligand of *Notch* (*N*), whereas ventral cells express another *N* ligand, *Delta* (*Dl*). *N* is active at both sides of the dorso-ventral (D/V) compartment boundary, and it is required to maintain the segregation of D and V cells [8]. Experiments that compared the behavior of cells mutant for *N* or *ap* at the D/V boundary [9]–[12] suggested that *ap* has an instructive role, and *N* a permissive one, in defining the D/V boundary [12], [13]. However, an alternative model proposed that N signaling is sufficient to separate D and V cells by creating a “fence” [10], [14], [15].

Segregation between distinct populations of cells also occurs in rhombomeres of the chick vertebrate hindbrain [16]. Rhombomeres have distinguishable cell lineages and express unique combinations of Hox genes [17], [18]. These genes specify the main axis in bilaterians [19], and are required to maintain the correct architecture of rhombomeres in the mouse hindbrain [20]. In *Drosophila*, experiments with imaginal discs in culture have shown that cells with different Hox addresses do not mix [21]–[23]. Moreover, an analysis carried out in the eye-antennal disc suggests that the Hox gene *Deformed* may be needed to establish a clonal restriction between maxillary and antennal fields [24]. However, the mechanism whereby Hox genes determine different cell affinities in the fly has not been addressed.

We show here that cells with different expression of the Hox gene *Ultrabithorax* segregate from each other and that this difference is sufficient to maintain A/P and D/V boundaries in the wing, haltere or leg disc. Differences in Ubx activity induce high levels of non-muscle myosin II. Other Hox genes seem to have a similar influence on myosin expression and compartment boundary maintenance. We propose that Hox genes may separate cells with different identity through the control of myosin accumulation.

## Materials and Methods

### Genetics

Most of the mutations and constructs are described in Flybase. Other constructs used are UAS-*dsUbx* [25], UAS-*OUbx* [26], *sqh*-GFP [27], *baz*-GFP and *zip*-GFP [28]. In the experiments with the Gal4/Gal80^ts^ system [29] the larvae were changed from 17°C to 29°C at the early third larval instar and kept at 29°C for 24h.

### Clonal Analysis

Clones of the following genotypes were induced at 24–48 h and 48–72 h (*smo*, *smo Ubx* and *Abd-B* clones) or 48–72 h and 72–96 h (*Ubx* clones) after egg laying with a one-hour heat-shock given at 37°C.

*y w hs*-flp122; FRT82B *Ubx^6.28^*/FRT82B *arm*-lacZ.

*y w hs*-flp122; *smo^3^* FRT40A *en*-lacZ/Ubi-GFP FRT40A.

*y w hs*-flp122; *smo^3^* FRT40A/Ubi-GFP FRT40A; *hh*-LacZ/+.

*y w hs*-flp122; *smo^3^* FRT40A *en*-lacZ/*smo^3^* FRT39; FRT82B *smo^+^* hs-GFP/FRT82B *Ubx^6.28^.*

*smo^3^* FRT40A/Ubi-GFP FRT40A; *bx^3^ hh*-lacZ/*TM2, Ubx^130.^*

*y w hs*-flp122; act>*y^+^*>Gal4/UAS-Ubx.

*sqh*-GFP/+; FRT82B *Ubx^6.28^*/FRT82B *arm*-lacZ.

*zip*-GFP/+; FRT82B *Ubx^6.28^*/FRT82B *arm*-lacZ.

*baz-GFP or baz*-GFP/+; FRT82B *Ubx^6.28^*/FRT82B *arm*-lacZ.

*sqh*-GFP/+; FRT82B *Abd-B^D18^*/FRT82B *arm*-lacZ.

To determine the crossing of the A/P boundary by *smo* mutant clones, each investigator scored the clones “blind”. Only in those cases in which the three researchers agreed we considered the clones as crossing or not crossing the compartment boundary.

### Immunochemistry

Antibody staining was done according to standard protocols. The antibodies used are: mouse anti-Ubx at 1/10 [30], rabbit anti-GFP (1∶200, Invitrogen), mouse anti-ß-galactosidase (1∶100, Cappel) and rabbit anti-ß-galactosidase (1∶2000, Cappel). TRITC-phalloïdine is from Sigma.

### Adult Cuticle Analysis

It was done following standard procedures.

## Results and Discussion

**Figure 1:**
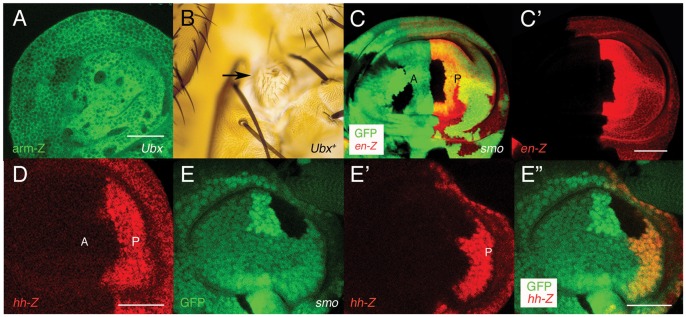
Hedgehog signaling and *Ultrabithorax* provide specific cell affinities to the cells. In Figures 1 and 2 anterior compartments (A) of the imaginal discs are to the left and posterior ones (P) to the right. (A) *Ubx* mutant clones, marked by the absence of *arm*-lacZ expression (in green), are round and tend to segregate from the surrounding tissue. (B) An *Ubx*-expressing clone (arrow), marked with *yellow* and induced in the second thoracic segment also segregates from the rest of the notum. (C, C′) A *smo* clone in the anterior compartment of the wing pouch, marked by the absence of GFP signal (in green), penetrates into the posterior compartment, which is marked by *en*-lacZ expression (in red). (D) *hh*-lacZ expression in the haltere disc. (E–E′′) A *smo* clone in the anterior compartment of the haltere pouch, marked as in C also penetrates into the posterior compartment, marked with *hh*-lacZ (in red, E′). Merged image in E′′. Scale bars are 30 µm in A, D, E′′ and 60 µm in C′.

The *Drosophila Ultrabithorax* (*Ubx*) Hox gene determines the development of the third thoracic segment (T3), and *Ubx* mutants transform this segment into the second thoracic one (T2) [31]. *Ubx* is expressed in the haltere discs, which will form the dorsal adult T3, but not in the wing disc (but for the peripodial membrane), which develops into the dorsal T2 [32]. As observed in the adult [33], cells lacking *Ubx* expression in the haltere disc do not mix with *Ubx*-expressing cells: *Ubx* mutant clones induced in this disc are round, with smooth borders and segregate from the surrounding epithelium (Fig. 1A). This segregation is also evident in clones expressing *Ubx* ectopically (Fig. 1B). These observations confirm that Ubx activity provides specific cell affinities.

### *Ultrabithorax* can Maintain the Antero-posterior Compartment Boundary in the Absence of Hh Signaling

Anterior clones mutant for *smo*, when abutting the A/P compartment boundary of the wing disc, frequently cross into the P compartment [6], [7] (Fig. 1C, C′). In the haltere disc, the A/P boundary is not so straight as in the wing disc (Fig. 1D), but we have also observed a similar behavior of many anterior *smo* clones (Fig. 1E–E′′ and Table 1; see also Fig.1d in Ref. 7).

**Table 1:** Number of clones crossing or respecting the A/P boundary in haltere, second and third leg disc in wildtype and mutant combinations.

Genotype	n	Cross	Do notcross	Othercases*
*smo^−^* in wildtype haltere disc	10	5	2	3
*smo^−^* in *bx^3^*/*TM2* haltere disc	12	0	7	5
*smo^−^* in wildtype II leg disc	18	0	11	6
*smo^−^* in wildtype III leg disc	11	6	1	4

* Small clones or clones where the crossing or not crossing was not evident.

**Figure 2:**
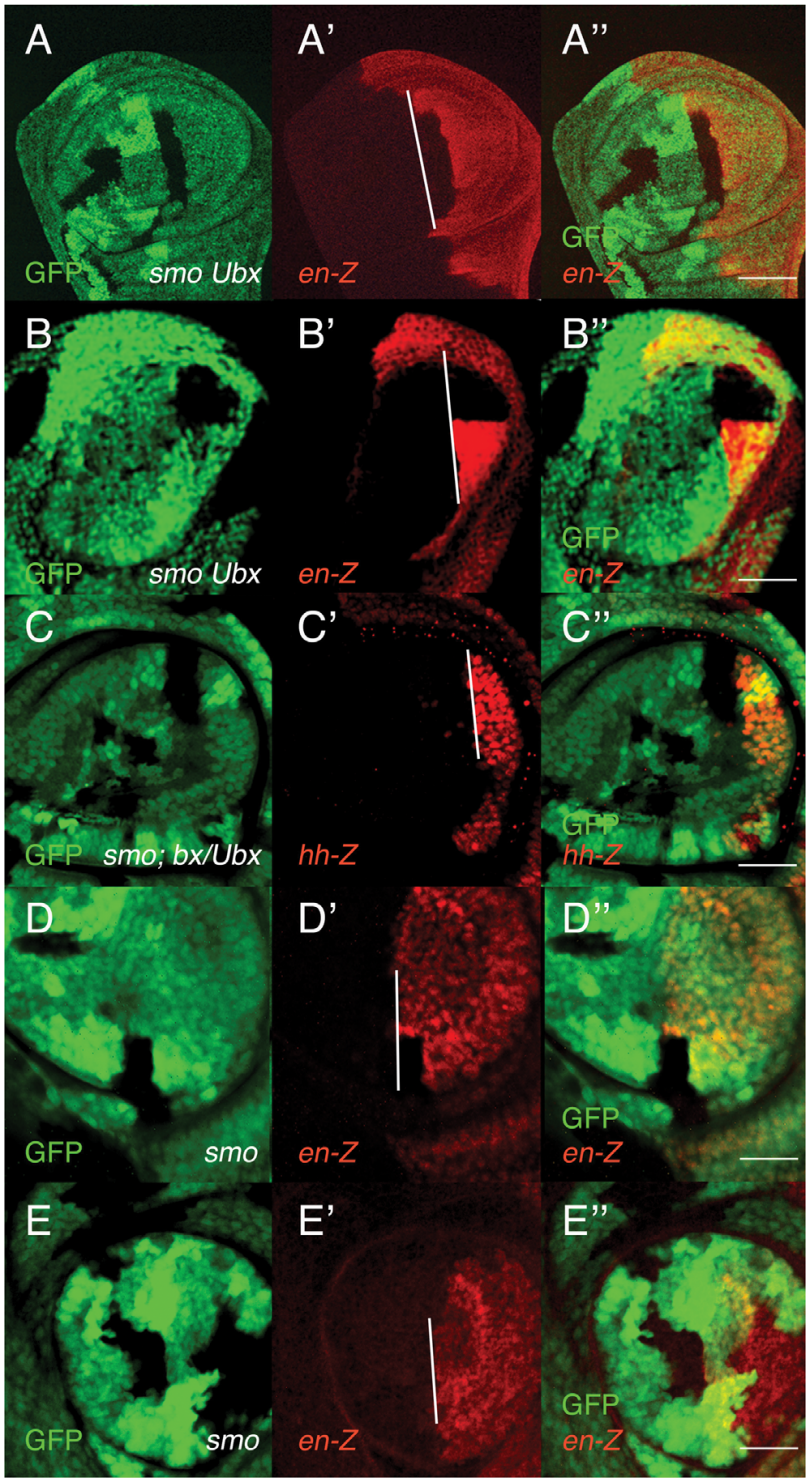
*Ultrabithorax* can maintain the A/P boundary in the absence of Hedgehog signaling. In all the panels of this Figure, the clones are marked by the lack of GFP in green, and the posterior compartment (to the right) is marked by either *hh*-lacZ or *en*-lacZ reporters in red. (A–B′′) Wing (A–A′′) and haltere (B–B′′) discs showing anterior clones double mutant for *smo* and *Ubx* that invade the posterior compartment. (C–C′′) *smo* clone induced in the anterior compartment of a *bx^3^/TM2, Ubx^130^* haltere disc. See that it does not cross the compartment boundary. Note that a few cells in the A compartment weakly express *hh*-lacZ. (D–D′′) An anterior *smo* clone induced in the third leg disc cross the A/P compartment boundary. (E–E′′) A similar clone induced in the second leg disc does not cross the boundary. Scale bars are 30 µm except in A′′ (60 µm).

The observation that either Ubx activity or Hh signaling can provide the cells with particular cell affinities, and that Hh is needed to maintain the separation of cells from A and P compartments, suggested the possibility that Ubx activity may be sufficient to maintain compartment boundaries. Anterior clones double mutant for *smo* and *Ubx*, if induced close to the A/P boundary of wing or haltere discs, can penetrate into the P compartment (Fig. 2A–B′′). This seems to suggest that differences in Ubx activity cannot compensate for the absence of *hh* signaling. However, in this experiment both A and P compartments express *Ubx*, and *Ubx^+^* cells from both compartments may equally reject the mutant cells. We wondered if a different activity of *Ubx* in A and P compartments could be sufficient to separate A and P cells when Hh signaling is compromised. To answer this question we induced anterior *smo^−^* clones in *bithorax (bx)* haltere discs, in which only the P cells express *Ubx* [31], [32]. These clones, when abutting the P compartment, respect the A/P boundary (Fig. 2C–C′′ and Table 1). The comparison of the distribution of *smo* clones induced in wildtype and *bx^−^* discs suggests that the different Ubx activity in A and P cells significantly contributes to maintain the A/P compartment boundary in the absence of Hh signaling.

We decided to study if the same result applies to the second and third leg discs. The rationale for these experiments is that *Ubx* is expressed in both compartments of the third leg disc but only in the P compartment of the second leg disc [34], [35]. The results presented in Fig. 2D–E′′ and in Table 1 show that most *smo^−^* clones induced in the A compartment of the third leg disc cross into the P compartment, whereas similar clones induced in the second leg disc respect the boundary. As also observed in the haltere disc, a few *smo^−^* clones in the third leg disc do not readily cross into the P compartment (Table 1). This may be due to Ubx being present at higher levels in the P compartment of this disc than in the A compartment [32], [36], or represent a coincidental event. Collectively, the data strongly suggest that *Ubx* can maintain the A/P boundary in the absence of Hh signaling.

### Ultrabithorax Maintains a Smooth Dorso-ventral Boundary in the Absence of Notch Signaling

**Figure 3:**
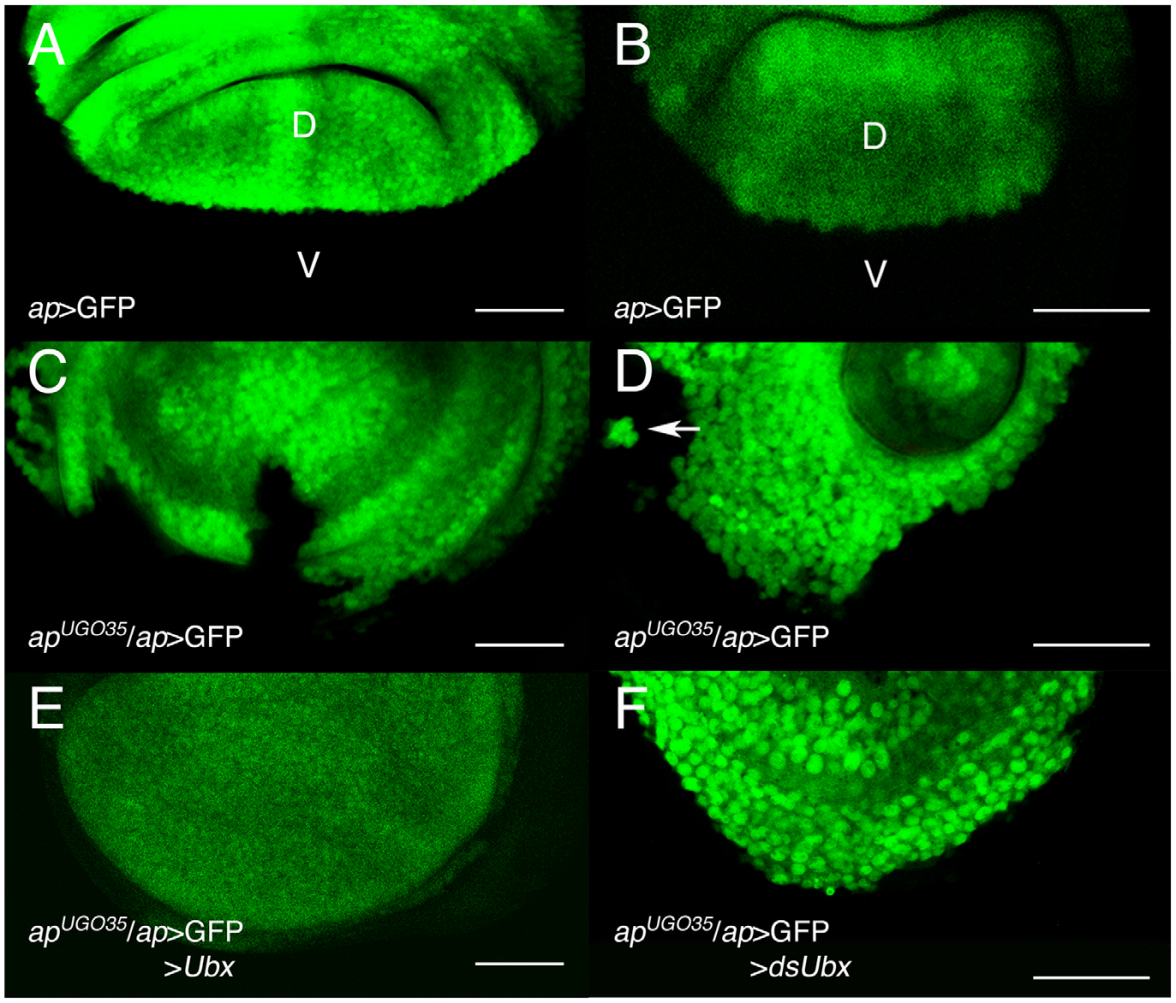
*Ultrabithorax* can maintain a smooth D/V boundary in the absence of *Notch* signaling. (A, B) *ap*-Gal4 UAS-GFP wing (A) and haltere (B) discs, showing the smooth boundary between dorsal (D, in green) and ventral (V) compartments. (C, D) In *ap*-Gal4 UAS-GFP/*ap^UGO35^* wing (C) and haltere (D) discs, this boundary is uneven. Note in D a group of dorsal cells in the ventral compartment (arrow). (E, F) In *ap*-Gal4 UAS-GFP/*ap^UGO35^*; UAS-*Ubx*/*tub-*Gal80^ts^ wing discs (E), or in haltere discs of *ap*-Gal4 UAS-GFP/*ap^UGO35^*; *Df109* UAS-*dsUbx*/*+* larvae (F), the straight D/V boundary is restored. See that the dorsal compartment in E is slightly reduced and that in F slightly enlarged. Scale bars are 40 µm in A, C and 30 µm in B, D, E and F.

Dorsal and ventral cells of the wing and haltere discs are separated by a straight D/V boundary (Fig. 3A, B). When *ap* expression is substantially reduced (*ap*-Gal4 UAS-GFP/*ap^UGO35^* discs), and N signaling, therefore, compromised, the boundary in the wing [12] (Fig. 3C) and haltere (Fig. 3D) disc is uneven. However, if we express *Ubx* in the dorsal compartment of an *ap^−^* wing disc (*ap*-Gal4 UAS-GFP/*ap^UGO35^*; UAS-*Ubx*/*tub-*Gal80^ts^ larvae; n = 14), so that there is Ubx activity only in the dorsal side, or if we reduce *Ubx* activity in the dorsal compartment of the haltere disc (*ap*-Gal4 UAS-GFP/*ap^UGO35^*; UAS-*dsUbx Df109*/*+* larvae; n = 11), and therefore keep *Ubx* activity only in the ventral compartment, the smooth D/V border is largely restored in both discs (Fig. 3E, F). These results suggest that strong differences in *Ubx* expression between dorsal and ventral compartments (that is, an on/off *Ubx* situation) can maintain a smooth D/V boundary in discs lacking N signaling.

### Differences in *Ultrabithorax* Expression Induce Accumulation of Myosin

It has been proposed that N signaling creates a “fence” that prevents cells from the D and V compartments from mixing [10], [14], [15]. Accordingly, elevated levels of F-actin and of the regulatory chain of non-muscle myosin II, encoded by the gene *spaghetti-squash* (*sqh*), are observed at this boundary in the wing disc [14], [15]. Significantly, a role for actomyosin in maintaining the A/P boundary in the wing disc [37] and in the embryo [38] has been described. Moreover, absence of *zipper* (*zip*), encoding the non-muscle myosin II heavy chain, prevents the maintenance of a normal D/V boundary in the wing disc [15].

**Figure 4:**
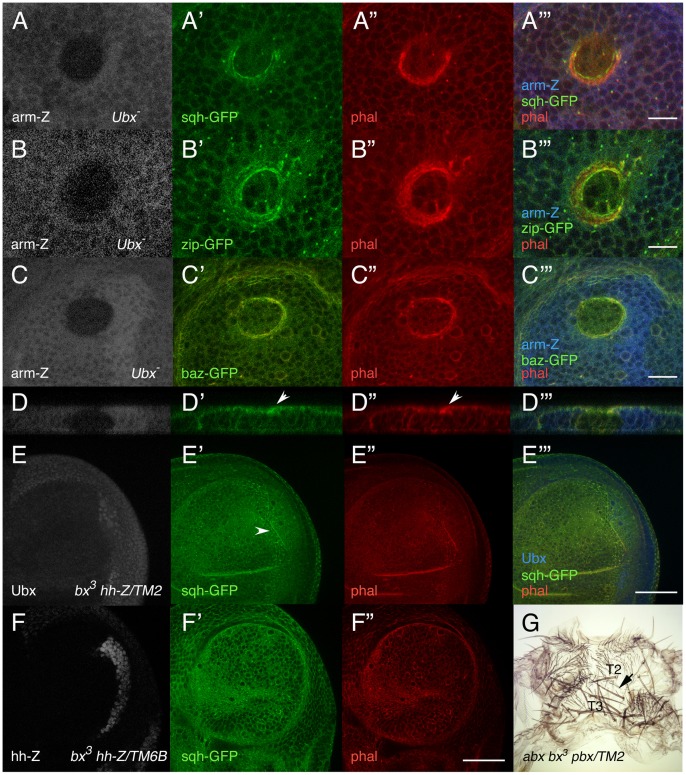
Differences in amount of *Ultrabithorax* between adjacent cells induce accumulation of *spaghetti-squash, zipper* and *bazooka.* (A–C′′′) Z-stacks of *Ubx* clones induced in the haltere disc, marked by the absence of *arm*-lacZ (in grey in A, B and C, in blue in A′′′, B′′′ and C′′′), showing a ring of *sqh*-GFP, *zip*-GFP or *baz*-GFP (in green in A′, B′ and C′, respectively), and higher levels of F-actin (in red in A′′, B′′ and C′′) around the clones. Merged images in A′′′, B′′′ and C′′′. In D–D′′′ we show a sagital section of the clone shown in C–C′′′. Note the invagination of the clone and the accumulation of *baz*-GFP and F-actin in the border of the clone (arrowheads). (E–E′′′) Haltere disc of the *bx^3^ hh*-lacZ/*TM2, Ubx^130^* genotype, showing accumulation of *sqh*-GFP (in green in E′, arrowhead) and F-actin (in red in E′′) at the A-P boundary, where compartments with (P compartment) and without (A compartment) *Ubx* abut (*Ubx* expression in grey in E and in blue in E′′′). Merged image in E′′′. (F–F′′) In *bx^3^ hh*-lacZ/*TM6B* haltere discs, by contrast, there is no accumulation of either *sqh*-GFP (in green in F′) or F-actin (in red in F′′) at the A-P boundary; *ß-galactosidase* expression is in grey in F. (G) *abx bx^3^ pbx*/*TM2, Ubx^130^* adult showing a fusion of the T2 and T3 (transformed into the T2) segments (arrow). Scale bars are 10 µm in A′′′, B′′′ and C′′′, and 30 µm in E′′′ and F′′.

To check if these proteins might also play a role in the separation of cells with different *Ubx* expression, we induced *Ubx* clones in the haltere disc and observed the expression of *sqh*-GFP and of *zip*-GFP. Most of these clones are surrounded by a ring of *sqh*-GFP, *zip*-GFP and phalloidine staining (Fig. 4A–B′′′). A lower level of *bazooka* (*baz*), a gene required to establish apico-basal cell polarity in *Drosophila* [39], was also reported at the wildtype D/V boundary of the wing disc [14], but in cells surrounding *Ubx* mutant clones *baz* expression is also increased (Fig. 4C–D′′′). Similar results are seen at the A/P compartment boundary of *bx^3^ hh-lacZ*/*TM2, Ubx^130^* haltere discs, in which only the A compartment lacks Ubx protein (Fig. 4E–E′′′). By contrast, in *bx^3^ hh-lacZ*/*TM6B* control discs the higher expression of *sqh*-GFP is not observed (Fig. 4F–F′′′). These results suggest that when the A and P compartments have different *Ubx* expression, the higher levels of myosin II at the *Ubx^−/^Ubx^+^* border may contribute to the maintenance of the A/P boundary in the absence of Hh signaling.

Although haltere and wing imaginal discs are physically separated throughout development, cells of different discs (as wing and haltere discs) form a continuous layer of adult cuticle during pupation. The different affinities in imaginal discs provided by Hox genes may prevent mixing of cells when this fusion takes place. In agreement with this idea, adult flies defective for *Ubx* occasionally present abnormal contralateral fusion of the T2 and T3 segments (Fig. 4G; G. Morata, personal communication).

### Different Hox Genes can Induce High *sqh* Levels

**Figure 5:**
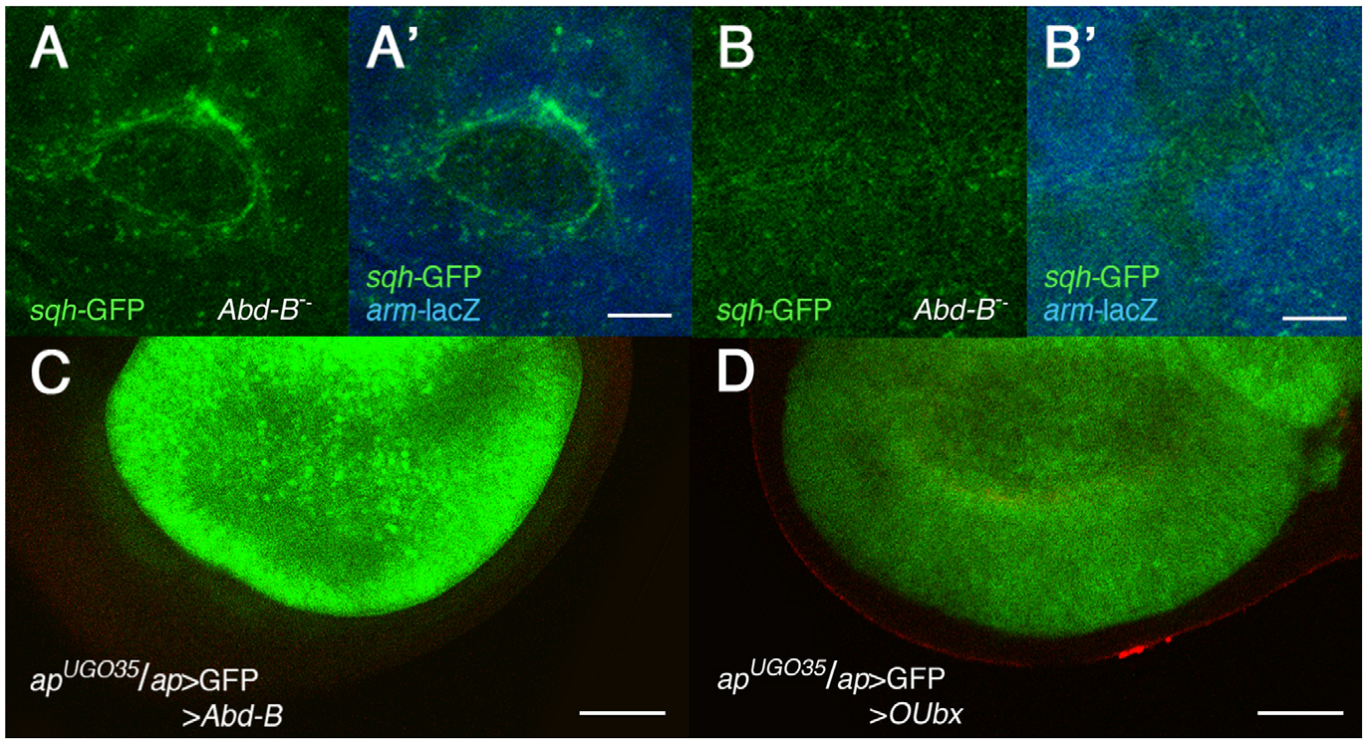
Differences in the amount of different Hox genes cause accumulation of *sqh*-GFP in imaginal discs. (A–A′′) Z-stack of an *Abd-B* mutant clone induced in the genital disc and marked by the absence of lacZ expression (in blue in A′) showing increased *sqh*-GFP expression around it (in green, A, A′). (B, B′) Z-stack of a control *Abd-B* clone similarly marked but induced in the wing disc, showing there is no increase in *sqh* levels. (C) In *ap*-Gal4 UAS-GFP/*ap^UGO35^*; UAS-*Abd-B*/*tub-*Gal80^ts^ wing discs, the D/V boundary is smooth (compare with Fig. 3C). (D) A similar result is obtained in *ap*-Gal4 UAS-GFP/*ap^UGO35^*; UAS-O*Ubx* **/** *tub-*Gal80^ts^.

To see if other Hox genes may also induce cell segregation, we induced *Abd-B* mutant clones and look for myosin expression in the genital disc, where this gene is required [40]. As shown in Fig. 5A, A′, increased expression of *sqh* is observed surrounding these clones, but not in control *Abd-B* clones induced in the wing disc, where *Abd-B* is not required (Fig. 5B, B′). Consistently with these results, the expression of *Abd-B* in the dorsal compartment of the wing pouch is sufficient to maintain a straight D/V boundary when *N* signaling is compromised (Fig. 5C; n = 11). Moreover, the expression of an onycophoran *Ubx* (UAS-*OUbx*) [26] is also sufficient to maintain this boundary (Fig. 5D; n = 12). Taken together, the results suggest that myosin may have a role in the segregation of cells with different Hox activity.

### Hox Genes and Cell Segregation

**Figure 6:**
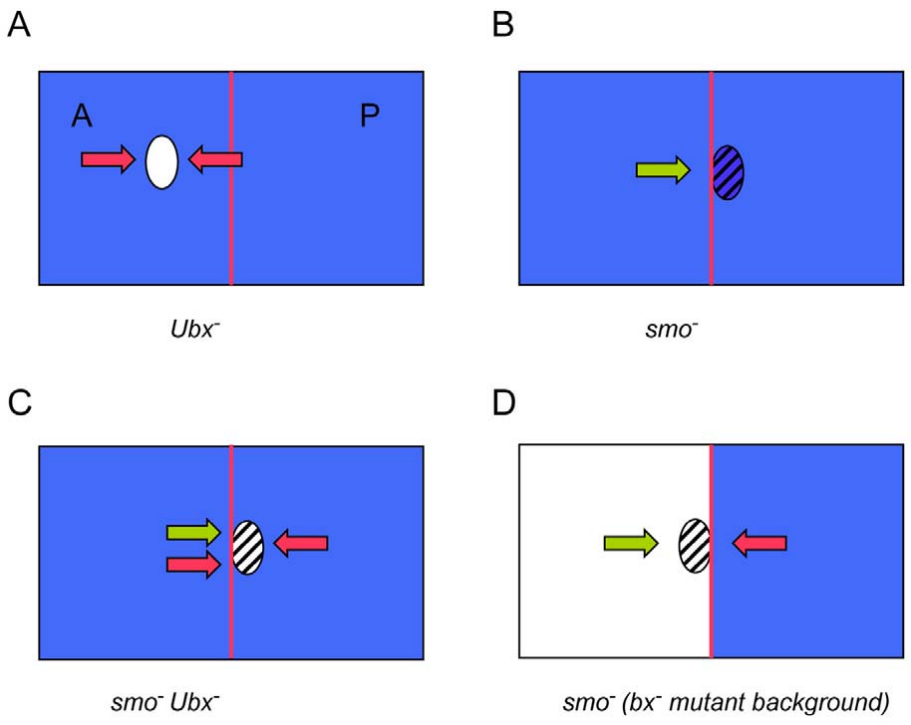
Summary of the results obtained with clones of different genotypes in the haltere disc. Anterior compartments are to the left, and the red line marks the A/P compartment boundary. *Ubx* expression is marked in blue and hatching indicates absence of Hh signaling. The red arrows indicate rejection of the cells of the clone due to different *Ubx* expression and the green arrows rejection due to different Hh signaling. (A) *Ubx^−^* clones are segregated from the rest of the tissue. (B) Anterior *smo* clones cross from the A to the P compartment. (C) Anterior *smo Ubx* clones undergo rejection from both A and P cells (because of their lack of *Ubx*) and rejection from A cells due to the absence of *smo*. The end result is the crossing of the boundary. (D) Clones like those in B, but induced in a *bx* background are rejected by both A and P cells and do not cross the boundary.

It was previously proposed that Hox genes confer different affinities to cells [41]. Studies in cultured *Drosophila* imaginal discs showed that cells from wildtype haltere and wing disc segregate, but that *Ubx* mutant haltere discs mix with wildtype wing discs [23]. We have shown here that *Ubx* is sufficient to maintain to a large extent the A/P and D/V boundaries in the absence of Hh and N signaling (see Fig. 6 for a summary of results). The mechanism whereby *Ubx* sorts out cells may be similar to that used by Hh and N signaling, and probably involves the accumulation of myosin where cells expressing and lacking *Ubx* are juxtaposed. This prevents territories with different properties to mix freely, and helps to get coherent patches of cells with distinct fates.

The sorting out of cells with distinct Hox activity in *Drosophila* has been reported before [24], [33], [40], [42]–[44], and in the case of the Hox gene *Deformed* a possible function in cell segregation has been assigned to such activity [24]. We have observed some cases that show that *Ubx* is needed to maintain segregation of cells from different segments during pupation. It is possible that *Drosophila* Hox genes may have a function in cell segregation during this pupal stage, where cells from different discs and histoblast nests fuse to develop the adult cuticle. The mechanism of segregation seems to rely on the confrontation of cells with different Hox function and not on the absolute levels of Hox expression. This implies that Hox activity in neighboring cells may be checked through proteins at the cell membrane whose expression or levels must be controlled by Hox genes. In the embryo, the Hox gene *Abd-B* has been shown to regulate molecules like cadherins [45], and such proteins may mediate segregation between adjacent cells with distinct Hox input.

In vertebrates, cells from different rhombomeres are also almost completely prevented from freely mixing [17]. As we have shown here for *Drosophila*, it has been proposed that the tension provided by the activity of actomyosin molecules, controlled by Hox genes, could prevent mixing of cells in the vertebrate’s rhombomeres [46]. Hox-directed cell segregation, therefore, prevents cells with different Hox code to intermingle, and therefore the appearance of homeotic transformations. This function of Hox genes may be an old one in evolution, required in animals in which development of different body regions is not coupled to the mechanisms of segmentation [47]. In *Drosophila*, this role of Hox genes may not be needed in cells that are physically separated during most of development (as in imaginal discs and histoblasts from different segments) or superseded by the activity of proteins like Engrailed and Hedgehog, but the maintenance of different affinities by Hox genes and signaling pathways through myosin accumulation may be a general mechanism to segregate cell populations in different species.
